# Evaluation and analysis of multidrug resistance- and hypervirulence-associated genes in carbapenem-resistant *Pseudomonas aeruginosa* strains among children in an area of China for five consecutive years

**DOI:** 10.3389/fmicb.2023.1280012

**Published:** 2023-10-12

**Authors:** Xin Zhang, Yunfen Zhu, Yuanyuan Gao, Wei Li, Yunzhong Wang, Yang Li

**Affiliations:** ^1^Department of Clinical Laboratory, Children’s Hospital of Soochow University, Suzhou, China; ^2^Institute of Pediatric Research, Children’s Hospital of Soochow University, Suzhou, Jiangsu, China; ^3^Clinical Medical College of Pediatrics, Soochow University, Suzhou, Jiangsu, China

**Keywords:** *Pseudomonas aeruginosa*, children, carbapenem resistance, hypervirulence, gene, type III secretion system, epidemiology

## Abstract

**Introduction:**

Carbapenem-resistant *Pseudomonas aeruginosa* (CRPA) is a growing threat. It is urgent to investigate the multidrug resistance and high virulence of CRPA to provide a basis for infection control and rational use of antibiotics.

**Methods:**

A retrospective study of 56 nonduplicated CRPA isolates was conducted.

**Results:**

CRPA mainly came from the intensive care unit (ICU) and was mostly isolated from sputum samples. The carbapenem resistance rates of *P. aeruginosa* were 21.37% (2016), 10.62, 5.88, 10 and 13.87% from 2016 to 2020, respectively. Carbapenem-resistant enzymes and aminoglycoside-modifying enzyme-encoding genes were detected in all isolates, and extended-spectrum β-lactamase and cephalosporin enzyme-encoding genes were present in 96.43 and 80.38% of isolates, respectively. The detection rate of *OprM* showed a statistically significant difference (*p* < 0.05) between the ICU and other wards. Genes related to biofilms, membrane channel proteins, I integrons and efflux systems were detected in all isolates, with detection rates greater than 90%. CRPA was strongly virulent, and over 80% of isolates carried hypervirulence-associated genes (*exoU*, *exoS*, *exoT*, and *exoY*). The drug resistance rates of cefepime and piperacillin/tazobactam showed a statistically significant difference (*p* < 0.05) between strains with *exoU* (+) and *exoU* (−) (*p* < 0.05). Notably, out of the 7 individuals who died, 4 had extensively drug-resistant *P. aeruginosa* (57.14%).

**Discussion:**

The detection rates of various resistance and virulence genes were high, and the coexistence phenomenon was serious. In clinical practice, antibiotics should be used reasonably based on different drug resistance genes to ensure the rationality and safety of patient medication.

## 1. Introduction

*Pseudomonas aeruginosa* (*P. aeruginosa*) is a common opportunistic pathogen in hospital-acquired infections. In 2017, the *World Health Organization* listed *P. aeruginosa* as a top priority pathogen. Many previous studies of *P. aeruginosa* infections have focused on adult patients. To date, there is less information about *P. aeruginosa* infections in children than in adults. *P. aeruginosa* infection usually has serious consequences for children, especially for those admitted to the intensive care unit (ICU). *P. aeruginosa* can be transmitted through multiple routes, causing infections in multiple parts of the human body, including the respiratory tract, circulatory system, urinary tract and wound surfaces (Hall et al., 2022). Antibiotic treatment is an effective method for treating *P. aeruginosa* infection. The introduction of antibiotics at the beginning of the 20th century revolutionized medicine, greatly reducing the incidence rate and mortality of bacterial infections. However, the increase in drug-resistant strains is occurring much more rapidly than the development of effective antibiotics. Due to the overuse of antibiotics, studies in various regions have shown an increase in drug resistance rates (Walkty et al., 2017; Sader et al., 2018; Lyu et al., 2023). Bacteria have been found to have severe resistance and are often multidrug resistant (MDR). At present, carbapenems are often the preferred antibiotics for terminal treatment of *P. aeruginosa* infections in clinical practice due to their broad antibacterial spectrum, strong antibacterial activity, and rapid action. However, the presence of carbapenem-resistant *Pseudomonas aeruginosa* (CRPA) isolates increases the incidence and mortality rates, length of hospital stay and treatment costs (Ponce de Leon et al., 2020). According to reports, the resistance of *P. aeruginosa* to carbapenems in other developing countries is approximately 50% (Sharifi et al., 2019). The latest data released by the *National Drug Resistance Monitoring Network*^1^ show that in the isolation rate of Gram-negative bacteria in hospitals in 2021, *P. aeruginosa* still ranked third, accounting for 11.8%. In terms of drug resistance, the national average resistance rate of *P. aeruginosa* to carbapenems is 17.7%, and the drug resistance rate in Jiangsu Province is higher than the national average level (24.3%). According to the CDC’s 2019 Antibiotic Resistance Threats Report, CRPA is listed as an urgent threat (CDC, 2019). According to the survey results of the *Chinese Children’s Bacterial Resistance Monitoring Group*, the resistance rate of *P. aeruginosa* to carbapenem antibiotics is rising rapidly, and it also has high resistance to other common antibiotics (Pan et al., 2021). With the widespread use of antibiotics, the numbers of multidrug-resistant *P. aeruginosa* (MDR-PA) and extensively drug-resistant *P. aeruginosa* (XDR-PA) strains are constantly increasing. However, very few antibiotics can effectively treat infections caused by MDR-PA and XDR-PA (Tamma et al., 2022). Therefore, it is of great importance to analyze the clinical distribution and drug resistance of *P. aeruginosa* for early antibiotic treatment.

At present, there are very limited effective antibiotics for CRPA, and a deep understanding of the resistance mechanism of CRPA is important for conducting new drug research and the rational clinical use of antibiotics. *P. aeruginosa* has both natural and acquired multidrug resistance, and its complex resistance mechanisms mainly include loss of outer membrane barrier pore protein (OprD_2_), overexpression of the efflux system, and production of β-lactam enzymes (BLEs) and aminoglycoside-modifying enzymes (AMEs). OprD_2_ is the only pore protein found in *P. aeruginosa* that is conducive to the passage of antibiotics (Chevalier et al., 2017). The downregulation of OprD_2_ usually leads to resistance to common carbapenem antibiotics (such as imipenem and meropenem). Therefore, the deletion or mutation of the gene encoding OprD_2_ can reduce drug uptake by *P. aeruginosa*. The efflux pumps are formed by a combination of *OprM*, which is found in the outer membrane, with *MexA* and *MexB* to form a stable complex at the inner membrane (Coșeriu et al., 2023). The most common efflux pump family in *Pseudomonas* is the resistance nodulation-division. *MexAB-OprM* plays an important role in the inherent resistance of *P. aeruginosa* (Ma et al., 2021). By overexpressing the MexAB-OprM complex, bacteria can acquire resistance to cephalosporins, penicillin, carbapenems, phenols, and most fluoroquinolones (Zahedi Bialvaei et al., 2021). The *MexR* gene is a regulator of the efflux pump *MexAB-OprM*. *MexA* and *MexB* are regulated by *MexR* on the operator of the efflux system. The production of BLEs is an important drug resistance mechanism of *P. aeruginosa*. BLEs include extended-spectrum β-lactamases (ESBLs), carbapenemases (MBLs), and cephalosporin enzymes (AmpC). ESBL production is an important mechanism of resistance of drug-resistant bacteria to penicillins, cephalosporins and aztreonam. MBLs are the most important drug resistance mechanism of CRPA (Elshafiee et al., 2019). Resistance to carbapenem antibiotics is mainly caused by Class B enzymes (Kim et al., 2016), and their resistance can be horizontally transmitted to other species. The genes encoding MBLs are usually part of the class I integron structure and are transmitted by mobile genetic elements (Palzkill, 2013). Integrins are a genetic structure that exists on bacterial plasmids, chromosomes, or transposons. They can capture drug resistance genes and make bacteria exhibit multiple-antibiotic resistance, which is also an important reason for the rapid development of bacterial multidrug resistance. Class I integrons represented by *intI-1* and *qacE*△*1-sul1* are the most common in clinical isolates. Whether *P. aeruginosa* can cause infection upon entering the body depends on two factors: one is the body’s defense ability, and the other is the pathogenic ability of the bacteria. Domestic and foreign studies have shown that the main pathogenesis of *P. aeruginosa* is closely related to its encoded polymorphic secretion system (Golovkine et al., 2018). The exoenzymes produced through the type III secretion system (T3SS) are an important virulence system for the pathogenicity of *P. aeruginosa*, which can lead to apoptosis of alveolar epithelial cells, macrophages, and fibroblasts, exacerbate inflammatory reactions, and increase the mortality rate of infection (Horna and Ruiz, 2021). Most *P. aeruginosa* have a functional T3SS, which contains virulence genes such as *exoS*, *exoT*, *exoU*, and *exoY*, which secrete effector proteins such as ExoS, ExoT, ExoU, and ExoY, respectively. Recently, a study showed that the detection rates of *exoA*, *exoS*, and *exoU* in *P. aeruginosa* were relatively high (Zarei et al., 2020), which poses great challenges to clinical treatment due to the widespread resistance of *P. aeruginosa*. The identification of virulence gene profiles is crucial for developing effective anti-*P. aeruginosa* infection strategies.

Due to the existence of multiple drug resistance mechanisms, the drug resistance rate of *P. aeruginosa* remains high, and it can secrete a large number of virulence factors, leading to a substantial increase in the incidence rate and mortality (Ponce de Leon et al., 2020). Exploring the coexistence of drug resistance genes and virulence genes is of great importance for the treatment of patients. Although there is currently information on the resistance and virulence genes of *P. aeruginosa*, the characteristics of various strains vary due to factors such as different targets of action, different sample sources, and different regions (Takata et al., 2018; de Sousa et al., 2023; Poursina et al., 2023). There are still few research papers and reviews on the correlation between CRPA infection in children in the Suzhou area. Therefore, we conducted a 5-year retrospective study to evaluate the epidemiological characteristics, resistance genes (*VIM*, *GIM*, *IMP*, *BIC*, *AIM*, *SPM*, *NDM*, *OXA*, *OXA-2*, *OXA-10*, *KPC*, *PER*, *VEB*, *SHV*, *TEM*, *CIT-1*, *MOX-1*, *EBC-1*, *ant(2″)-I*, *ant(2″)-Ia*, *ant(3″)-I*, *aac(3)-II*, *aac(3)-IIc*, *ant(4′)-Ia*, *aac(6′)-I*, *aac(6′)-Ib*, *aac(6′)-II*, *OprM*, *MexB*, *mucB*, *intI-1*, *qacE*△*1-sul1*, *OprD_2_*) and hypervirulence-associated genes (*exoU*, *exoS*, *exoT* and *exoY*) of *P. aeruginosa* infection in children in the Suzhou area to explore the reasons for the resistance and high virulence of CRPA at the molecular level and promote the rational use of antibiotics in clinical practice based on the detected virulence and resistance gene carriers.

## 2. Materials and methods

### 2.1. Study site

This study was conducted at the Children’s Hospital of Soochow University (CHSU), which is a children’s medical center in East China and the only provincial tertiary children’s hospital in Jiangsu Province. CHSU has 1,500 beds and serves >70,000 inpatients and > 2 million outpatients annually.

### 2.2. General information

From January 2016 to December 2020, a total of 82 CRPA strains were isolated, of which 56 nonduplicated CRPA strains were selected as the research subjects. *P. aeruginosa* isolates were confirmed by mass spectrometry (Microflex LT/SH, Germany). An automatic bacterial detection and analysis system (VITEK^®^2 Compact, France) and Kirby-Bauer (KB) method were used for the drug sensitivity test. The results were analyzed according to the guidelines of the Clinical and Laboratory Standards Institute. The quality control strains were *P. aeruginosa* (ATCC 27853) which were purchased from the clinical testing center of the National Health Commission. This study was approved by the ethics committee of CHSU (No. 2021CS158).

### 2.3. Determination of drug-resistant *Pseudomonas aeruginosa*

The judgment criteria of CRPA were *P. aeruginosa* resistant to imipenem and one of the following eight categories of antibiotics, including cephalosporin (such as ceftazidime and cefepime), quinolones (such as levofloxacin and ciprofloxacin), carbapenem (such as imipenem and meropenem), β-lactam enzyme inhibitors (such as piperacillin/tazobactam and cefoperazone/sulbactam), aminoglycosides (such as gentamicin, tobramycin, and amikacin), monobactams (aztreonam), phosphates (fosfomycin), and polymyxin (polymyxin B). Resistant to 3 and more than three antibiotics are considered MDR, resistant to 6 and more than six are considered XDR, and strains showing resistance to all the tested drugs are considered pan-drug resistant (Magiorakos et al., 2012).

### 2.4. Detection of virulence and resistance genes

Focusing on the analysis of the resistance and virulence genes, specific gene sequences can be found in Supplementary Table S1. Bacterial DNA was extracted by the boiling method, as described in the literature (Wei et al., 2007). The specific steps were as follows: a ring of colonies was picked from the inoculation ring, placed in an EP tube containing 1 mL of sterile water, shaken and mixed evenly. Then, the colonies were boiled in a 100°C metal bath for 30 min and cooled in a −20°C refrigerator for 30 min to lyse the cells, completely thawed at room temperature, and centrifuged at 15000 rpm for 5 min. The supernatant was collected and stored. After measuring its concentration and purity by UV spectrophotometry, it was placed in a refrigerator at −20°C as a template for storage.

### 2.5. Data analysis

SPSS 20.0 and WHONET v5.6 software (WHO Collaborating Centre for Surveillance of Antimicrobial Resistance, Boston, MA, USA) were used to analyze the data. The counting data were expressed as the number of cases (n) and rate (%). The *χ*^2^ test was used in univariate analysis. The comparison between groups was carried out by the *χ*^2^ test, with *p* < 0.05 indicating a statistically significant difference.

## 3. Results

### 3.1. Clinical information analysis of 56 CRPA strains

A total of 56 CRPA isolates were collected. Among them, 33 strains were isolated from the ICU, 39 isolates were isolated from sputum, and 7 strains were isolated from deceased patients. The source of the specimen showed a statistically significant difference (*p* < 0.05) between the ICU and other wards. The probability of patients admitted to the ICU being infected with other pathogens was significantly higher than that of patients in other wards (Table 1).

**Table 1:** Epidemiology in this study [n (%)].

Classification		ICU (*n* = 33)	Other wards^*^ (*n* = 23)	*χ^2^*	*P*
Specimen	Sputum (*n* = 39)	27 (69.23)	12 (30.77)	**5.634**	**0.018**
	Blood (*n* = 4)	1 (25)	3 (75)	2.049	0.152
	Alveolarlavage fluid (*n* = 5)	4 (80)	1 (20)	1.007	0.316
	Urine (*n* = 4)	0 (0)	4 (100)	**6.181**	**0.013**
	Others specimen^*^ (*n* = 4)	1 (25)	3 (75)	2.049	0.152
Age	0 ~ <3Y (*n* = 39)	24 (61.54)	15 (38.46)	0.362	0.548
	3 ~ <14Y (*n* = 16)	9 (56.25)	7 (43.75)	0.066	0.797
	14 ~ <18Y (*n* = 1)	0 (0)	1 (100)	1.461	0.227
Survival state	Survival (*n* = 49)	27 (55.10)	22 (44.90)	2.372	0.124
	Death (*n* = 7)	6 (85.71)	1 (14.29)		
Gender	Male (*n* = 36)	20 (55.56)	16 (44.44)	0.474	0.491
	Female (*n* = 20)	13 (65)	7 (35)		
Length of hospitalization	0 ~ <15 d (*n* = 8)	4 (50)	4 (50)	0.307	0.579
	15 ~ <30 d (*n* = 11)	4 (36.36)	7 (63.64)	2.880	0.090
	30 ~ <60 d (*n* = 25)	17 (68)	8 (32)	1.536	0.215
	> = 60 d (*n* = 12)	8 (66.67)	4 (33.33)	0.378	0.539
Basic disease	Pneumonia (*n* = 20)	11 (55)	9 (45)	0.198	0.656
	Hematological system diseases (*n* = 14)	7 (50)	7 (50)	0.615	0.433
	Heart disease (*n* = 12)	10 (83.33)	2 (16.67)	3.758	0.053
	Other infections^*^ (*n* = 5)	2 (40)	3 (60)	0.813	0.367
	Other diseases^*^ (*n* = 5)	3 (60)	2 (40)	0.003	0.959
Any combined infection with other pathogenic bacteria	Gram-positive bacteria (*n* = 7)	4 (57.14)	3 (42.86)	0.011	0.918
	Gram-negative bacteria (*n* = 25)	12 (48)	13 (52)	2.229	0.135
	Mixed infection of Gram-negative/positive bacteria or fungi (*n* = 11)	11 (100)	0 (0)	**9.541**	**0.002**
	No combined infection (*n* = 13)	6 (46.15)	7 (53.85)	1.142	0.285

Bold values highlight meaningful results. ^*^Other specimens included pharyngeal swabs and pus. Other infections included encephalitis, laryngitis, urinary tract infection, and lymphadenitis. Other diseases included epilepsy, genetic metabolic disease, foreign body inhalation asphyxia, and neonatal respiratory distress syndrome. Other wards included the departments of hematology, neonatology, and respiratory care. d, days; Y, years.

### 3.2. The detection and carbapenem-resistance rate of *Pseudomonas aeruginosa*

**Figure 1:**
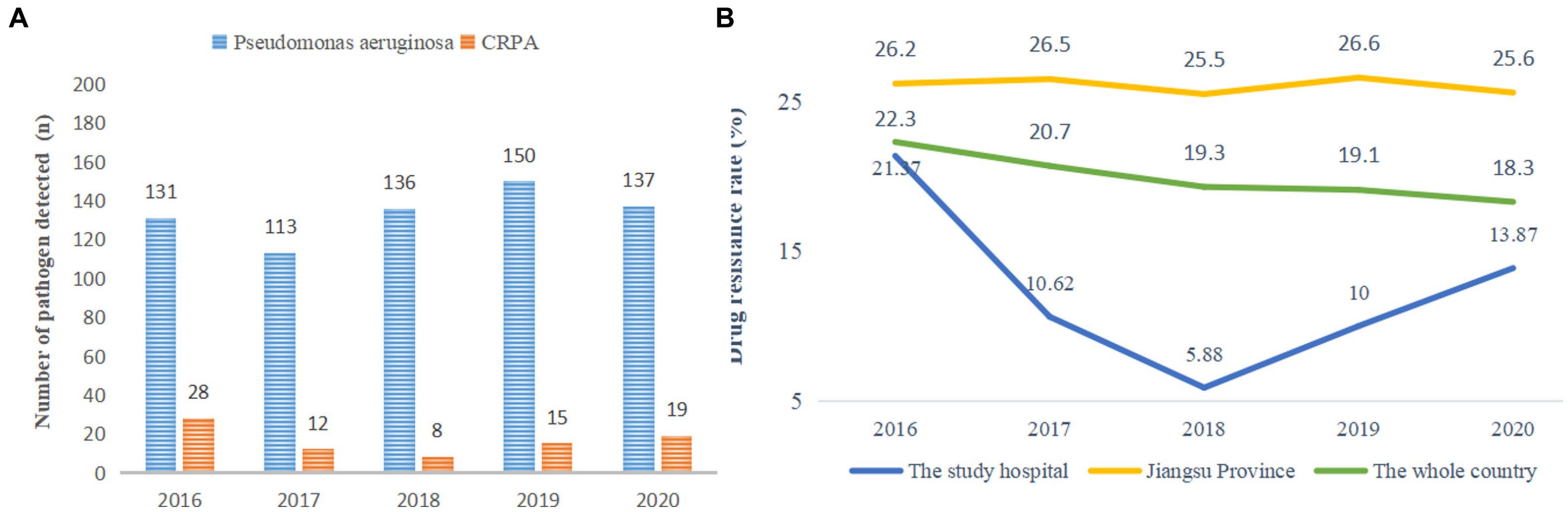
**(A)** Detection of pathogenic bacteria. **(B)** Carbapenem resistance rate of *P. aeruginosa* from 2016 to 2020. * The carbapenem resistance rate of *P. aeruginosa* in the whole country came from the National Drug Resistance Monitoring Network. The member units that report the data are mainly tertiary hospitals, which report the monitoring data from October of the current year to September of the next year every year. The principle of retaining the first strain of the same bacteria from the same patient is to eliminate duplicate strains.

In this study, the detection of *P. aeruginosa* fluctuated between 2016 and 2020, with the lowest amount of CRPA isolates detected in 2018 (Figure 1A). The carbapenem resistance rates of *P. aeruginosa* were 21.37, 10.62, 5.88, 10 and 13.87% from 2016 to 2020, respectively. The carbapenem resistance rate in the local children’s medical center was significantly lower than that in Jiangsu Province and the whole country of China (Figure 1B).

### 3.3. Analysis of antimicrobial susceptibility

A total of 56 CRPA isolates exhibited a higher drug resistance rate to various antibiotics but fluoroquinolone antibiotics, as shown in Table 2. They showed a 100% resistance rate to carbapenems (imipenem), extended-spectrum cephalosporins (cefotetan, ceftriaxone), enzyme inhibitor complexes (ampicillin/sulbactam), penicillins (ampicillin), and sulfonamides (compound sulfamethoxazole). The resistance rate to fluoroquinolones (ciprofloxacin and levofloxacin) was only 7.14%.

**Table 2:** Analysis of the drug resistance of 56 CRPA isolates.

Antibiotic		Number of drug-resistant bacteria	Percentage (%)
Carbapenems	Imipenem	56	100
Monobactams	Aztreonam	28	50
Extended-spectrum cephalosporin	Cefotetan	56	100
	Ceftazidime	18	32.14
	Ceftriaxone	56	100
	Cefepime	9	16.07
Penicillins	Ampicillin	56	100
	Piperacillin	20	35.71
	Ticarcillin	40	71.43
Enzyme inhibitor complexes	Ampicillin/sulbactam	56	100
	Piperacillin/tazobactam	17	30.36
	Cefoperazone/sulbactam	18	32.14
Aminoglycosides	Gentamicin	0	0
	Amikacin	0	0
	Tobramycin	0	0
Sulfonamides	Compoundsulfamethoxazole	56	100
Fluoroquinolones	Ciprofloxacin	4	7.14
	Levofloxacin	4	7.14

### 3.4. Detection of BLEs genes in 56 CRPA isolates

BLEs genes include carbapenem resistance genes, ESBLs genes and AmpC genes. In Table 3, the detection rates of *VIM*, *OXA*, *GIM*, *BIC* and *IMP* were 98.21, 57.14, 55.36, 30.36, and 17.86%, respectively. NDM, *AIM*, *SPM*, *KPC*, *OXA-2* and *OXA-10* were not detected. See Supplementary Table S2 for the details of resistance genes carried by the 56 isolates.

**Table 3:** The results of BLEs gene detection in 56 CRPA isolates [n (%)].

Gene	Type	Number of isolates with detected genes			*χ^2^*	*P*
		Total	ICU (*n* = 33)	Other wards^*^ (*n* = 23)		
Carbapenem-resistant genes	*VIM*	55 (98.21)	32 (57.14)	23 (41.07)	0.71	0.4
	*OXA*	32 (57.14)	18 (32.14)	14 (25)	0.221	0.638
	*GIM*	31 (55.36)	17 (30.36)	14 (25)	0.48	0.488
	*BIC*	17 (30.36)	10 (17.86)	7 (12.5)	0	0.992
	*IMP*	16 (17.86)	10 (17.86)	6 (10.71)	0.118	0.731
	*AIM*	0	0	0	–	–
	*SPM*	0	0	0	–	–
	*NDM*	0	0	0	–	–
	*KPC*	0	0	0	–	–
	*OXA-2*	0	0	0	–	–
	*OXA-10*	0	0	0	–	–
ESBLs genes	*SHV*	54 (96.43)	31 (55.36)	23 (41.07)	1.446	0.229
	*VEB*	0	0	0	–	–
	*PER*	0	0	0	–	–
	*TEM*	0	0	0	–	–
AmpC genes	*EBC-1*	43 (76.79)	25 (44.65)	18 (32.14)	0.048	0.827
	*CIT-1*	32 (57.14)	22 (39.29)	10 (17.86)	2.976	0.085
	*MOX-1*	0	0	0	–	–

^*^Other wards included the departments of hematology, neonatology, and respiratory care.

### 3.5. Detection of other main genes of the 56 CRPA isolates

AMEs genes and other resistance genes were analyzed. As seen in Table 4, *ant(2″)-I*, *mucB*, *OprD_2_*, *intI-1*, *qacE*△*1-sul1*, and *MexB* were detected in all isolates. See Supplementary Table S2 for the details of resistance genes carried by the 56 isolates.

**Table 4:** The results of other main gene detection in 56 CRPA isolates [n (%)].

Gene	Type	Number of isolates with detected genes			*χ^2^*	*P*
		Total	ICU (*n* = 33)	Other wards^*^ (*n* = 23)		
AMEs genes	*ant(2″)-I*	56 (100)	33 (58.93)	23 (41.07)	–	–
	*aac(6′)-II*	48 (85.71)	27 (48.21)	21 (37.5)	0.996	0.318
	*aac(3)-IIc*	35 (62.5)	21 (37.5)	14 (25)	0.044	0.833
	*ant(2″)-Ia*	30 (53.57)	18 (32.14)	12 (21.43)	0.031	0.861
	*aac(6′)-Ib*	28 (50)	20 (35.71)	8 (14.29)	3.615	0.057
	*aac(6′)-I*	6 (10.71)	4 (7.14)	2 (3.57)	0.166	0.683
	*ant(4′)-Ia*	0	0	0	–	–
	*ant(3″)-I*	0	0	0	–	–
	*aac(3)-II*	0	0	0	–	–
Biofilm-related genes	*mucB*	56 (100)	33 (58.93)	23 (41.07)	–	–
Membrane channel protein-related genes	*OprD_2_*	56 (100)	33 (58.93)	23 (41.07)	–	–
I integron	*intI-1*	56 (100)	33 (58.93)	23 (41.07)	–	–
	*qacE*△*1-sul1*	56 (100)	33 (58.93)	23 (41.07)	–	–
Genes related to the efflux system	*MexB*	56 (100)	33 (58.93)	23 (41.07)	–	–
	*OprM*	52 (92.86)	33 (58.93)	19 (33.93)	**6.181**	**0.013**

Bold values highlight meaningful results. ^*^Other wards included the departments of hematology, neonatology, and respiratory care.

### 3.6. Detection of hypervirulence-associated genes of 56 CRPA isolates

In Tables 4, 5 hypervirulence-associated genes were analyzed. *exoS, exoT, and exoY* were present in 100% of the isolates. In addition, 83.93% of the strains carried *exoU*. See Supplementary Table S3 for the details of hypervirulence-associated genes carried by the 56 isolates.

**Table 5:** The results of virulence gene detection in 56 CRPA isolates [n (%)].

Hypervirulence-associated genes	Number of isolates with detected genes			*χ^2^*	*P*
	Total	ICU (*n* = 33)	Other wards^*^ (*n* = 23)		
*exoU*	47 (83.93)	26 (55.32)	21 (44.68)	1.574	0.210
*exoS*	56 (100)	33 (58.93)	23 (41.07)	–	–
*exoT*	56 (100)	33 (58.93)	23 (41.07)	–	–
*exoY*	56 (100)	33 (58.93)	23 (41.07)	–	–

^*^Other wards included the departments of hematology, neonatology, and respiratory care.

### 3.7. The correlation between carrying exoU and antibiotic resistance of 56 CRPA isolates

As shown in Table 6, there were differences in cefepime and piperacillin/tazobactam resistance among strains carrying *exoU*, while there was no difference in other antibiotics.

**Table 6:** Correlation analysis between the resistance rate and *exoU* [n (%)].

Antibiotics	Number of drug resistant bacteria		*χ^2^*	*P*
	*exoU* (+)* (*n* = 47)	*exoU* (−) (*n* = 9)		
Imipenem	47 (83.93)	9 (16.07)	–	–
Aztreonam	25 (44.64)	3 (5.36)	1.191	0.275
Cefotetan	47 (83.93)	9 (16.07)	–	–
Ceftazidime	17 (30.36)	1 (1.78)	2.175	0.140
Ceftriaxone	47 (83.93)	9 (16.07)	–	–
Cefepime	9 (16.07)	0	**8.640**	**0.003**
Ampicillin	47 (83.93)	9 (16.07)	–	–
Piperacillin	18 (32.14)	2 (3.57)	0.850	0.356
Ticarcillin	35 (62.5)	5 (8.93)	1.324	0.250
Ampicillin/sulbactam	47 (83.93)	9 (16.07)	–	–
Piperacillin/tazobactam	17 (30.36)	0	**4.674**	**0.031**
Cefoperazone/sulbactam	16 (28.57)	2 (3.57)	0.484	0.487
Gentamicin	0	0	–	–
Amikacin	0	0	–	–
Tobramycin	0	0	–	–
Compoundsulfamethoxazole	47 (83.93)	9 (16.07)	–	–
Ciprofloxacin	4 (7.14)	0	0.825	0.364
Levofloxacin	4 (7.14)	0	0.825	0.364

Bold values highlight meaningful results. ^*^exoU (+) represents the detection of this gene.

### 3.8. Differential analysis between the survival group and death groups

Comparison of data between the survival and death groups showed that there was no statistically significant difference in the detection rates of major drug resistance genes or hypervirulence-associated genes (Table 7). Notably, out of the 7 individuals who died, 4 had XDR bacteria detected.

**Table 7:** Related comparative analysis of the survival group and death group [n (%)].

Classification		Survival group (*n* = 49)	Death group (*n* = 7)	*χ^2^*	*P*
Carbapenem-resistant genes	*VIM* (*n* = 55)	48 (87.27)	7 (12.73)	0.145	0.703
	*OXA* (*n* = 32)	27 (84.38)	5 (15.62)	0.667	0.414
	*GIM* (*n* = 31)	27 (87.1)	4 (12.9)	0.01	0.919
	*BIC* (*n* = 17)	16 (94.12)	1 (5.88)	0.977	0.323
	*IMP* (*n* = 16)	12 (75)	4 (25)	3.200	0.074
ESBLs genes	*SHV* (*n* = 54)	47 (87.04)	7 (12.96)	0.296	0.586
AmpC genes	*EBC-1* (*n* = 43)	37 (86.05)	6 (13.95)	0.358	0.550
	*CIT-1* (*n* = 32)	27 (84.38)	5 (15.62)	0.667	0.414
AMEs genes	*aac(6′)-II* (*n* = 48)	42 (87.5)	6 (12.5)	0	1
	*aac(3)-IIc* (*n* = 35)	30 (85.71)	5 (14.29)	0.27	0.602
	*ant(2″)-Ia* (*n* = 30)	26 (86.67)	4 (13.33)	0.041	0.839
	*aac(6′)-Ib* (*n* = 28)	23 (82.14)	5 (17.86)	1.469	0.225
	*aac(6′)-I* (*n* = 6)	5 (83.33)	1 (16.67)	0.107	0.744
Genes related to the efflux system	*OprM* (*n* = 52)	45 (86.54)	7 (13.46)	0.615	0.433
Hypervirulence-associated genes	*exoU* (+) (*n* = 47)	41 (87.23)	6 (12.77)	0.019	0.891
Any combined infection with other pathogenic bacteria	Gram-positive bacteria (*n* = 7)	7 (100)	0 (0)	1.143	0.285
	Gram-negative bacteria (*n* = 25)	23 (92)	2 (8)	0.836	0.361
	Mixed infection of Gram-negative/positive bacteria or fungi (*n* = 11)	8 (72.73)	3 (27.27)	2.731	0.098
	No combined infection (*n* = 13)	11 (84.62)	2 (15.38)	0.129	0.720
Basic disease	Pneumonia (*n* = 20)	19 (95)	1 (5)	1.600	0.206
	Hematological system diseases (*n* = 14)	10 (71.43)	4 (28.57)	**4.408**	**0.036**
	Heart disease (*n* = 12)	11 (91.67)	1 (8.33)	0.242	0.622
	Other infections^*^ (*n* = 5)	5 (100)	0 (0)	0.784	0.376
	Other diseases^*^ (*n* = 5)	4 (80)	1 (20)	0.282	0.595

Bold values highlight meaningful results. ^*^exoU (+) represents the detection of this gene. Other infections included encephalitis, laryngitis, urinary tract infection, and lymphadenitis. Other diseases included epilepsy, genetic metabolic disease, foreign body inhalation asphyxia, and neonatal respiratory distress syndrome.

## 4. Discussion

*Pseudomonas aeruginosa* can rapidly mutate and acquire resistance to adapt to the environment, making it one of the main pathogens causing hospital infections. Carbapenem antibiotics are currently the “ace killer” of Gram-negative bacteria due to their strong selectivity and low toxicity to host cells. However, with the widespread use of antibiotics, the detection rate of *P. aeruginosa* resistant to multiple antibiotics is increasing yearly (Litwin et al., 2020). The emergence of CRPA has made hospital infection control increasingly difficult. CRPA has a high mortality rate, is prone to cross-infection, and is extremely difficult to treat. Its drug resistance genes are easily transmitted between strains, leading to the production of drug-resistant bacteria and thus affecting treatment effectiveness. In addition, very few antibiotics can effectively treat infections caused by multidrug-resistant *P. aeruginosa* (Wanqing et al., 2022). The emergence, prevention, and treatment of hospital infections and new drug-resistant bacteria are extremely important.

In the present work, 56 CRPA strains mainly came from three key departments: the ICU, neonatal pediatrics, and hematology. The proportion of CRPA originating from the ICU exceeded 50%, which may be related to the rapid spread of CRPA among ICU patients. This result is consistent with a recent survey on CRPA infections in critically ill children in a large tertiary pediatric hospital in China (Huang et al., 2023). The incidence of infection among ICU patients was 5-7-fold higher than that among general inpatients, contributing to 20–25% of all nosocomial infections in hospitals (Alfouzan et al., 2021). Subsequently, statistical analysis was conducted on the types of specimens, and it was found that most CRPA isolates were detected in sputum specimens, accounting for 69.64%, which is consistent with reports that CRPA is mainly isolated from sputum (Gill et al., 2022). There was a statistically significant difference in the detection of CRPA detected in sputum and urine between the ICU and other wards. From an age group perspective, detection was mainly concentrated among children aged 0 ~ <3 years. The proportion of CRPA isolated from males was slightly higher than that isolated from females. Patients who had been hospitalized for more than 30 days had a higher proportion of CRPA detection (66.07%). Analysis of 7 CRPA strains isolated from deceased children found that 6 strains originated from sputum and 1 strain from blood. The mortality rate in the sputum sample group was 15.38% (6/39), while in the blood group, the mortality rate was 25% (1/4). The mortality rate of bloodstream infections caused by CRPA is high. Special attention should be given to CRPA detected in the blood circulation system (Recio et al., 2020). Among the clinical cases corresponding to the 56 CRPA strains, pneumonia, hematological system diseases, and heart disease accounted for the highest proportion of diseases. Children admitted to the ICU were more likely to develop mixed infections (*p* = 0.002). In response to the upstream situation, clinical practitioners should pay attention to high-risk factors, take timely response measures, isolate and protect, and perform early prevention, detection, and treatment.

To further explore the detection of *P. aeruginosa* and the discovery of carbapenem antibiotic resistance, from 2016 to 2020, the carbapenem resistance rate of *P. aeruginosa* showed a downward and then upward trend, which was consistent with a previous report (Feng et al., 2021) and far lower than the drug resistance rate shown in another study (Sharifi et al., 2019). However, the drug resistance rate continued to increase from 2018 to 2020, which was inconsistent with the downward trend reported by the *National Drug Resistance Monitoring Network* (*CHINET*) (see text footnote 1) (Hu et al., 2021). The resistance of *P. aeruginosa* to imipenem is primarily caused by the production of metalloenzymes and efflux pumps and the lack of OprD_2_ (Memar et al., 2021), which may be related to the widespread use of imipenem in clinical practice in recent years, and resistance is gradually increasing. According to feedback from the *Chinese Children’s Bacterial Resistance Monitoring Group*, the detection rate of CRPA in 2020 was 9.5% (Leiyan et al., 2021), lower than the 13.87% in this study and the national rate (18.3%). This indicates that the drug resistance rate of *P. aeruginosa* in children’s hospitals is still relatively high. If hypervirulence-associated genes are combined, it will be tremendously difficult to make diagnoses and provide treatment. Therefore, special attention needs to be paid to the infection situation of CRPA in children.

Exploring the drug resistance mechanism of CRPA and analyzing its high toxicity and pathogenicity need to be performed. Subsequently, the related resistance genes (including production of BLEs and AMEs, loss of OprD_2_ and overexpression of the efflux system) and hypervirulence-associated genes were analyzed. The PCR results indicated that among 56 CRPA isolates, the proportion of BLE-producing strains was quite high. Carbapenem resistance genes were detected in 56 strains. The most prevalent gene detected in this study was *VIM*, in agreement with other study (Gill et al., 2022). There is evidence that the rapid dissemination of MBL-producing *P. aeruginosa* is mainly due to *VIM* and *NDM* (Urbanowicz et al., 2019). Very few studies have reported on the coexistence of VIM and NDM in the same bacterium (Jaggi et al., 2012). Regarding differences in drug resistance genes detected in different regions, different types of carbapenem resistance (*IMP, NDM, DIM* and so on) among *P. aeruginosa* strains have been detected in Europe (Paul et al., 2016). ESBLs genes were detected in 54 strains, and AmpC genes were detected in 45 strains. ESBL-producing Gram-negative pathogens are a major cause of resistance to ESBL antibiotics. In the past, *TEM* and *SHV*-type ESBLs were the predominant families of ESBLs. In this work, *SHV* was the only main ESBL gene, accounting for 96.43%. *VEB, PER and TEM* were not detected. Currently, *CTX-M*-type enzymes are the most commonly found ESBLs type (Castanheira et al., 2021). AmpC enzyme is a type of β-lactam enzyme mediated by chromosomes or plasmids, including *ACC*, *DHA*, *CIT*, *EBC*, *FOX* and *MOX*. As shown in Table 2, the detection rates of *EBC-1, CIT-1* and *MOX-1* were 76.79, 57.14% and 0, respectively. This is somewhat different from the results reported in other studies (Miao et al., 2019), indicating that CRPA from different regions and sample sources may have different drug resistance rates and drug resistance gene carriers.

*Pseudomonas aeruginosa* forms drug resistance by producing AMEs. Reportedly, *aac(6′)-I*, *aac(6′)-II*, *ant(2″)-I* and *aph(3′)* are the most common AMEs genes in *P. aeruginosa* (Asghar and Ahmed, 2018). According to the report by Cabrera, the *aac(3)-Ia, aac(3)-Ic, aac(6″)-Ib* and *ant(2″)-Ia* genes were associated with aminoglycoside-resistant strains (Cabrera et al., 2022). The results showed that *aac(6′)-Ib* was the most commonly (26/43, 60.4%) identified AME-encoding gene (Ahmadian et al., 2021). A total of 11 AMEs genes were analyzed in 56 isolates of CRPA. *ant(2″)-I* plays an important role in aminoglycoside-resistant antibiotics. The PCR results showed that *MexB* was present in 100% of the 56 isolates. Comparative analysis of *OprM* gene detection between the ICU and other departments found that the gene was detected in all 33 CRPA strains isolated from the ICU, while only 19 CRPA strains from other departments had the gene, with a statistically significant difference (*p* = 0.013). The *MexAB-OprM* efflux pump system can transport β-lactams, quinolones and macrolides, causing *P. aeruginosa* to have a wide range of drug resistance (Henrichfreise et al., 2007). There is a need for vigilance of the spread of drug-resistant bacteria in ICU wards. Other resistance genes (*mucB*, *OprD_2_*, *intI-1*, *qacE*△*1-sul1*) were present in 100% of the 56 CRPA isolates. These above results indicate that the resistance of 56 strains of CRPA was closely related to the formation of efflux pump systems and biofilms, suggesting the development of corresponding technologies or drugs from these aspects to improve the effectiveness of clinical treatment of CRPA.

In addition to the existence of multiple drug resistance genes, a large number of virulence factors also significantly increase the invasion and pathogenicity of the bacterium, leading to an increase in the incidence rate and mortality (Takata et al., 2018). A report by Omid et al. showed that the detection rate of hypervirulence-associated genes in *P. aeruginosa* isolates is high, and multidrug-resistant and highly virulent *P. aeruginosa* poses great challenges to clinical treatment (Zarei et al., 2020). The identification of virulence gene profiles is crucial for developing effective anti-*P. aeruginosa* infection strategies. The exozymes produced by the type III secretion system (T3SS) are important virulence systems for the pathogenicity of *P. aeruginosa*. Therefore, analyzing whether patients carry corresponding virulence genes will help clinicians focus on the patient’s condition and prevent serious clinical symptoms leading to death. Some studies have shown that *exoU* gene-positive *P. aeruginosa* shows higher multidrug resistance and more significant mortality than other T3SS gene-expressing *P. aeruginosa* (Takata et al., 2018; Khodayary et al., 2019). The results showed that *exoS*, *exoT* and *exoY* were present in all isolates, except for *exoU* (83.93%). Further analysis of *exoU* toxicity showed that a total of 12 children had CRPA detected in at least two specimens, of which 11 children had CRPA carrying *exoU*, accounting for 91.67% (11/12). This indicates that strains carrying *exoU* have strong virulence and are prone to penetrating cells and invading other parts, leading to infection. The coexistence of resistance and high virulence factors in CRPA is an alarming threat. This finding underlines the importance of monitoring multidrug resistance to determine optimal therapeutic options against such infections.

The correlation between the drug resistance rate of 56 CRPA strains and the *exoU* gene was further analyzed. The results of the drug sensitivity test showed that the 56 CRPA isolates exhibited a higher drug resistance rate to various antibiotics, but it was lower than that in a recent study that analyzed CRPA isolated from children (Huang et al., 2023). The drug resistance rates of cefepime and piperacillin/tazobactam were related to whether they carried *exoU*, and the difference was statistically significant (*p* < 0.05). *P. aeruginosa* has natural resistance to many kinds of antibiotics and easily develops acquired resistance in the course of treatment, so a combination of drugs is the first choice for the clinical treatment of *P. aeruginosa*. In addition, this study found that the resistance rate of 56 CRPA strains to fluoroquinolones (ciprofloxacin and levofloxacin) was only 7.14%, and the resistance rate to aminoglycoside antibiotics, such as amikacin, tobramycin, and gentamicin, was 0. The abovementioned drugs have a relatively high degree of harm to children and are less commonly used in clinical practice (Lin et al., 2016), resulting in a lower resistance rate, which is consistent with the expected results. AMEs genes are associated with resistance to multiple antibiotics (Atassi et al., 2023). Combined with the analysis of genetic testing results, the detection rate of AMEs genes and *MexB* was high, while the resistance rate of corresponding aminoglycoside antibiotics was 0, which suggests that there may be potential resistance factors, and the relationship between resistance genes and antibiotics needs to be further explored. In view of this, carbapenem drugs or combination drugs should be used reasonably in the treatment of CRPA infections, such as β-lactam in combination with aminoglycosides or β-lactam combined with fluoroquinolones, but the dosage should be reasonably controlled. The drug sensitivity results suggest that it is necessary to strengthen the monitoring of bacterial resistance in clinical practice and actively promote and guide the rational use of antibiotics.

To determine the risk factors for CRPA infection in children, a retrospective case–control study was conducted that included 56 patients with CRPA infection. This study consisted of 56 MDR-PA strains and 31 XDR-PA strains. The clinical outcome of children infected with CRPA was poor, with a mortality rate of 12.5%, which is lower than the mortality rate reported by Lodise et al. (2022) (20.0% ~ 30.8%). Comparison of data between the survival and death groups showed that there was no statistically significant difference in the detection rates of major drug resistance genes or hypervirulence-associated genes. Strains carrying the *exoU* are more virulent, as they can encode and produce a strong toxin protein, which can lead to cell necrosis and apoptosis (Zhu et al., 2023). There was no statistically significant difference in the detection of an exoU gene between the survival group and the death group. Patients with hematological system diseases are themselves a high-risk population infected with carbapenem-resistant Gram-negative bacteria (Zhao et al., 2020). Among the 7 deceased children, 4 children suffered from hematological diseases. Comparing the data on hematological diseases between the survival group and the death group, the difference was statistically significant (*p* = 0.036). An interesting finding of this study is that out of the 7 deceased children, 4 children had isolated XDR-PA, accounting for 57.14% (4/7). Special attention needs to be paid to the detection of MDR-PA and XDR-PA and timely control of infection to avoid exacerbating the condition of children.

In summary, CRPA, as an important nosocomial infectious pathogen, was mainly isolated from sputum and ICU patients. CRPA has gradually increased in drug resistance and virulence, making treatment more difficult. The coexistence of resistance and high virulence factors in CRPA is an alarming threat. Analyzing the multiple drug resistance- and hypervirulence-associated genes carried by CRPA can help clinicians gain a deeper understanding of the coexistence mechanism of drug resistance and virulence genes. However, there are some limitations in this study. Due to limited testing conditions, only representative resistance- and hypervirulence-associated genes were selected, resulting in limited clinical value. In future research, it is necessary to collect more strains and expand the scope of gene analysis, conduct in-depth research on the resistance mechanism and virulence of CRPA, and understand the correlation between multidrug resistance and high virulence.

## Data availability statement

The datasets used and/or analyzed during the current study available from the corresponding author on reasonable request.

## Ethics statement

The studies involving humans were approved by the Medical Ethics Committee of the Children’s Hospital of Soochow University (ethics batch number: 2021CS158). The studies were conducted in accordance with the local legislation and institutional requirements. Written informed consent for participation was not required from the participants or the participants’ legal guardians/next of kin because simple retrospective analysis of data does not involve patient privacy.

## Author contributions

XZ: Conceptualization, Data curation, Formal analysis, Methodology, Writing – original draft. YZ: Conceptualization, Formal analysis, Methodology, Writing – original draft, Writing – review & editing. YG: Data curation, Formal analysis, Writing – original draft. WL: Data curation, Methodology, Writing – original draft. YW: Conceptualization, Formal analysis, Writing – review & editing. YL: Conceptualization, Funding acquisition, Writing – review & editing, Formal analysis.

## Footnotes

^1^ http://www.carss.cn/
