# Supplementary material for: Evaluation and analysis of multidrug resistance- and hypervirulence-associated genes in carbapenem-resistant Pseudomonas aeruginosa strains among children in an area of China for five consecutive years
Source: Front Microbiol. 2023 Oct 12;14:1280012. doi: 10.3389/fmicb.2023.1280012 (PMC10602738; doi:10.3389/fmicb.2023.1280012)
Supplement: Supplementary file 1 [file Table_1.docx]

**Supplementary Material 1**

Genes includes BLEs genes (*VIM, GIM, IMP, BIC, AIM, SPM, NDM, OXA, OXA-2, OXA-10, KPC, PER, VEB, SHV, TEM, CIT-1, MOX-1, EBC-1*)^[1-3]^, AMEs genes (*ant(2″)-Ⅰ, ant(2″)-Ⅰa, ant(3″)-Ⅰ, aac(3)-Ⅱ, aac(3)-Ⅱc, ant(4')-Ⅰa, aac(6')-Ⅰ, aac(6')-Ⅰb, aac(6')-Ⅱ*)^[4-5]^, other resistance genes (*OprM, MexB, mucB, intⅠ-1, qacE△1-sul1, OprD_2_*)^[6]^, high virulence genes (*exoU, exoY, exoS* and *exoT*)^[7-8]^. Detailed gene sequence information is shown in Table S1. The gene sequence in the experiment was synthesized by Shanghai Branch of Beijing Qingke Biotechnology Co., Ltd. Polymerase chain reaction (PCR) was used to detect the carrier of target genes in the isolates. The PCR products were analysed by 2% agarose gel electrophoresis and positive products were sequenced and aligned with sequences in GenBank using the Basic Local Alignment Search Tool.

Table S1 Gene sequence

| Gene | | Sequence（5'-3'） | Size of product (bp) |
| --- | --- | --- | --- |
| AMEs genes | *ant(2″)-Ⅰ* | F:GCGCGAAATCTGCCGCTCTGG | 320 |
|  |  | R:CTGTTACAACGGACTGGCCGC |  |
|  | *ant(2″)-Ⅰa* | F:GCTCACGCAACTGGTCCAGA | 719 |
|  |  | R:GGCACGCAAGACCTCAACCT |  |
|  | *ant(3″)-Ⅰ* | F:TGATTTGCTGGTTACGGTGAC | 284 |
|  |  | R:CGCTATGTTCTCTTGCTTTTG |  |
|  | *aac(3)-Ⅱ* | F:ACTGTGATGGGATACGCGTC | 237 |
|  |  | R:CTCCGTCAGCGTTTCAGCTA |  |
|  | *aac(3)-Ⅱc* | F:ACGCGGAAGGCAATAACGGA | 854 |
|  |  | R:TAACCTGAAGGCTCGCAAGA |  |
|  | *ant(4')-Ⅰa* | F:CTGCTAAATCGGTAGAAGC | 172 |
|  |  | R:CAGACCAATCAACATGGCACC |  |
|  | *aac(6')-Ⅰ* | F:TATGAGTGGCTAAATCGA | 394 |
|  |  | R:CCCGCTTTCTCGTAGCA |  |
|  | *aac(6')-Ⅰb* | F:CATGACCTTGCGATGCTCTA | 490 |
|  |  | R:GCTCGAATGCCTGGCGTCTT |  |
|  | *aac(6')-Ⅱ* | F:TTCATGTCCGCGAGCACCCC | 178 |
|  |  | R:GACTCTTCCGCCATCGCTCT |  |
| ESBLs genes | *PER* | F:AGTCAGCGGCTTAGATA | 978 |
|  |  | R:CGTATGAAAAGGACAATC |  |
|  | *VEB* | F:GCGGTAATTTAACCAGA | 961 |
|  |  | R:GCCTATGAGCCAGTGTT |  |
|  | *SHV* | F:AAGCGAAAGCCAGCTGTCG | 176 |
|  |  | R:TTCGCTCCAGCTGTTCGTC |  |
|  | *TEM* | F:TCGGGGAAATGTGCG | 440 |
|  |  | R:TGCTTAATCAGTGAGGCACC |  |
| Carbapenem-resistant genes | *OXA-2* | F:CGCTGTTCGTGATGAGTTCC | 210 |
|  |  | R:ATCGGCGTTGCCATAGTC |  |
|  | *OXA* | F:GCGTGGTTAAGGATGAACAC | 438 |
|  |  | R:CATCAAGTTCAACCCAACCG |  |
|  | *KPC* | F:CGTCTAGTTCTGCTGTCTTG | 798 |
|  |  | R:CTTGTCATCCTTGTTAGGCG |  |
|  | *NDM* | F:GGTTTGGCGATCTGGTTTTC | 621 |
|  |  | R:CGGAATGGCTCATCACGATC |  |
|  | *VIM* | F:GTGGATCGGGTTGTAGTCG | 215 |
|  |  | R:CCACCTTCAGCATCGTCA |  |
|  | *IMP* | F:GAAGGCGTTTATGTTCATAC | 587 |
|  |  | R:GTAAGTTTCAAGAGTGATGC |  |
|  | *GIM* | F:TCGACACACCTTGGTCTGAA | 432 |
|  |  | R:TCATTGGCGGTGCCGTACAC |  |
|  | *AIM* | F:CTGAAGGTGTACGGAAACAC | 445 |
|  |  | R:GTTCGGCCACCTCGAATTG |  |
|  | *SPM* | F:TATGCAGCTCCTTTAAGGGC | 271 |
|  |  | R:ACATTATCCGCTGGAACAGG |  |
|  | *OXA-10* | F:TCCGAGTATGCCGTAAATG | 822 |
|  |  | R:GCTCTTGTTTGGACCGCTAT |  |
|  | *BIM* | F:TATGCAGCTCCTTTAAGGGC | 508 |
|  |  | R:TCATTGGCGGTGCCGTACAC |  |
| AmpC genes | *MOX-1* | F:GCTGCTCAAGGAGCACAGGAT | 520 |
|  |  | R:CACATTGACATAGGTGTGGTGC |  |
|  | *CIT-1* | F:TGGCCAGAACTGACAGGCAAA | 462 |
|  |  | R:TTTCTCCTGAACGTGGCTGGC |  |
|  | *EBC-1* | F:TCGGTAAAGCCGATGTTGCGG | 302 |
|  |  | R:CTTCCACTGCGGCTGCCAGTT |  |
| Other resistant genes | *OprM* | F:ATTCTGGTCCTGGGCTACC | 328 |
|  |  | R:CTGGGAACTCGATCTCTTCG |  |
|  | *MexB* | F:TAACCGTCGGGATTGACCT | 202 |
|  |  | R:CCAAGCTGAACAGCTACCAG |  |
|  | *mucB* | F:CGATGAACACCGAGAATCGT | 274 |
|  |  | R:CTGAACGAGAAGGGGCAGT |  |
|  | *intⅠ-1* | F:CCGAGGATGCGAACCACTTC | 373 |
|  |  | R:CCGCCACTGCGCCGTTACCA |  |
|  | *qacE△1-sul1* | F:TAGCGAGGGCTTTACTAAGC | 300 |
|  |  | R:ATTCAGAATGCCGAACACCG |  |
|  | *OprD_2_* | F:ACGCGATTTGACGGTGGT | 188 |
|  |  | R:GCATCTCCAAGACCATGCTG |  |
| Hypervirulence-associated genes | *exoU* | F:GATCTTATTTCGCTGCTCGA | 1038 |
|  |  | R:CCTTCTGGCGAAAAGCCAC |  |
|  | *exoS* | F:GCGAGGTCAGCAGAGTATCG | 118 |
|  |  | R:TTCGGCGTCACTGTGGATGC |  |
|  | *exoY* | F:CGGATTCTATGGCAGGGAGG | 289 |
|  |  | R:GCCCTTGATGCACTCGACCA |  |
|  | *exoT* | F:AATCGCCGTCCAACTGCATGCG | 152 |
|  |  | R:TGTTCGCCGAGGTACTGCTC |  |

Reference:

1. Ahmed N, Ali Z, Riaz M, et al. Evaluation of Antibiotic Resistance and Virulence Genes among Clinical Isolates of *Pseudomonas aeruginosa* from Cancer Patients [J]. Asian Pac J Cancer Prev. 2020, 21(5): 1333-1338.
2. YT Jiang, JL Mai, DQ Chen, et al. Detection and analysis of drug resistance Genetic testing of Carbapenem resistant Pseudomonas aeruginosa [J]. Chinese Journal of Antibiotics, 2016, 41 (7): 552-556.
3. LZ Zhou, JD Wang, XM Qian, et al. Study on the Plasmid AmpC Gene of Continuous Isolates of Escherichia coli and Klebsiella pneumoniae [J]. Modern Practical Medicine, 2006, 18 (11): 777-779.
4. SH Sun, BZ Zhuge, DQ Zhu, et al. Detection of aminoglycoside modifying enzyme Genetic testing of multi drug resistant mucoid Pseudomonas aeruginosa [J]. Chinese Journal of Microbiology, 2019, 31 (11): 1269-1273.
5. F Zhang, P Guo, LJ Zhang, et al. Genetic testing of Aminoglycoside resistance in Pseudomonas aeruginosa [J]. Journal of Anhui Medical University, 2018, 53 (6): 928-932.
6. ZH Si, WJ Lin, SQ Xu, et al. Detection of drug resistance Genetic testing of Pseudomonas aeruginosa in primary hospitals [J]. Chinese Journal of Hospital Infection, 2017, 27 (7): 1448-1451.
7. YT Jiang, HX Ouyang, AW Wu, et al. The correlation between virulence genes and drug resistance of multidrug-resistant Pseudomonas aeruginosa [J]. Journal of Tropical Medicine, 2016, 16 (5): 604-607.
8. Dacheux D, Toussaint B, Richard M, et al. Pseudomonas aerugino-sa cystic fibrosis isolates induce rapid type l secretion-dependent but exoU-independent, oncosis of macrophages and polymorphonucle ar neutrophil [J]. Infect Immun, 2000, 68 (5): 2916-2924.
